# 20-Hydroxyeicosatetraenoic Acid Impairs Endothelial Insulin Signaling by Inducing Phosphorylation of the Insulin Receptor Substrate-1 at Ser^616^

**DOI:** 10.1371/journal.pone.0095841

**Published:** 2014-04-24

**Authors:** Xuguang Li, Gang Zhao, Ben Ma, Rui Li, Jiang Hong, Shaowen Liu, Dao Wen Wang

**Affiliations:** 1 Department of Cardiology, Shanghai First People's Hospital, School of Medicine, Shanghai Jiao Tong University, Shanghai, People's Republic of China; 2 Department of Internal Medicine, Tongji Hospital, Tongji Medical College, Huazhong University of Science and Technology, Wuhan, People's Republic of China; H.Lee Moffitt Cancer Center & Research Institute, United States of America

## Abstract

20-hydroxyeicosatetraenoic acid (20-HETE) induces endothelial dysfunction and is correlated with diabetes. This study was designed to investigate the effects of 20-HETE on endothelial insulin signaling.Human umbilical vein endothelial cells (HUVECs) or C57BL/6J mice were treated with 20-HETE in the presence or absence of insulin, and p-ERK1/2, p-JNK, IRS-1/PI3K/AKT/eNOS pathway, were examined in endothelial cells and aortas by immunoblotting. eNOS activity and nitric oxide production were measured. 20-HETE increased ERK1/2 phosphorylation and IRS-1 phosphorylation at Ser^616^; these effects were reversed by ERK1/2 inhibition. We further observed that 20-HETE treatment resulted in impaired insulin-stimulated IRS-1 phosphorylation at Tyr^632^ and subsequent PI3-kinase/Akt activation. Furthermore, 20-HETE treatment blocked insulin-stimulated phosphorylation of eNOS at the stimulatory Ser^1177^ site, eNOS activation and NO production; these effects were reversed by inhibiting ERK1/2. Treatment of C57BL/6J mice with 20-HETE resulted in ERK1/2 activation and impaired insulin-dependent activation of the IRS-1/PI3K/Akt/eNOS pathway in the aorta. Our data suggest that the 20-HETE activation of IRS-1 phosphorylation at Ser^616^ is dependent on ERK1/2 and leads to impaired insulin-stimulated vasodilator effects that are mediated by the IRS-1/PI3K/AKT/eNOS pathway.

## Introduction

Insulin promotes vasorelaxation and capillary recruitment in peripheral tissues and is a potent pro-angiogenic molecule that regulates neovascularization and EC migration [1]. These functions are mediated by a signaling cascade involving insulin receptor substrate-1 (IRS-1), PI3-kinase (PI3K), Akt, endothelial NO synthase (eNOS), and the generation of NO [2]. Accumulating experimental evidence suggests that both endothelial NO synthase (eNOS) and nitric oxide (NO) are involved in the pathogenesis of diabetes and insulin resistance [3]. Associated micro- and macro-vascular complications of metabolic disorders (e.g., retinopathy, nephropathy, hypertension, atherosclerosis, and coronary artery disease) are preceded by a state of endothelial dysfunction that is characterized by impaired NO bioavailability and vasorelaxation. Altered eNOS expression, NO production, and endothelial dysfunction, are important features of insulin-resistant conditions and diabetes [4].

The synthesis of 20-hydroxyeicosatetraenoic acid (20-HETE), the ω-hydroxylation product of arachidonic acid, is catalyzed by enzymes of the cytochrome P450 (P450) 4 gene family (CYP4A, -4B, and -4F) [5]. Increased 20-HETE levels have been observed in pathological conditions including ischemic cerebrovascular diseases, cardiac ischemia-reperfusion injury, kidney diseases, hypertension, and diabetes mellitus [6]–[8]. Evidence has accumulated that suggests a role for 20-HETE in vascular disorders such as atherosclerosis and hypertension [9]–[13], which are associated with endothelial dysfunction and insulin resistance. Moreover, the 20-HETE inhibitor HET0016 attenuated the development of diabetes-induced vascular dysfunction [14]–[16]. These findings indicate that the effects of 20-HETE on the development of hypertension and vascular dysfunction constitute the mechanisms by which 20-HETE contributes to endothelial dysfunction in diabetes and other insulin-resistant conditions.

Furthermore, evidence suggests that 20-HETE activates the mitogen-activated protein kinase (MAPK) pathway by stimulating ERK1/2 phosphorylation in endothelial cells [17]. Several serine residues in IRS-1 have been identified as negative regulatory sites, including Ser^616^ (orthologous to Ser^612^ in rat IRS-1), which is activated by mitogen-activated protein kinase (MAPK) [18]. It remains unclear, however, whether activation of this kinase by 20-HETE would affect the phosphorylation of IRS-1 at Ser^616^, thus impairing activation of the insulin vasodilatory signaling pathway involving PI 3-kinase/Akt/eNOS.

The present study investigated whether 20-HETE affects insulin signaling that involves the production of NO in endothelial cells.

## Materials and Methods

### Materials

L-[^14^C]arginine and L-[^14^C]citrulline were obtained from PerkinElmer Inc. (Santa Clara, CA, USA). Antibodies for phospho-ERK1/2, ERK1/2, phospho-JNK, JNK, IRS-1, phospho-IRS-1 (Ser^312^), phospho-IRS-1 (Ser^616^), phospho-IRS-1 (Ser^612^), phospho-IRS-1 (Tyr^632^), phospho-IRS-1 (Tyr^628^), the p85 subunit of PI3K, phospho-protein kinase B (AKT; Ser^473^), AKT, eNOS, phospho-eNOS(Ser^1177^), and β-actin were obtained from Santa Cruz Biotechnology (Santa Cruz, CA, USA). PD98059 (a reversible MEK1 inhibitor), SP600125 (inhibitor of JNK), *N* ^G^-nitro-L-arginine (L-NNA, inhibitor of NOS), cGMP ELISA kit and 20-Hydroxyeicosatetraenoic acid (20-HETE) were obtained from the Cayman Chemical Company (Ann Arbor, MI, USA). Other reagents were obtained from Sigma (St. Louis, MO, USA).

### Endothelial Cell Culture

Human umbilical vein endothelial cells(HUVECs) were obtained from Lonza, and were cultured according to the manufacturer's instruction. The ECM was consisted of 10% fetal bovine serum, 1% endothelial cell growth supplement.

### Animals

C57BL/6J mice(6 weeks old) were obtained from Shanghai Experimental Animal Center, Chinese academy of science. All animals were maintained in a pathogen-free environment and given radiation-sterilized food pellets and distilled water. Experiments were carried out according to the National Institutes of Health Guide for Care and Use of Laboratory Animals and were approved by the Bioethics Committee of Shanghai Jiao Tong University. All surgery was performed under sodium pentobarbital anesthesia, and all efforts were made to minimize suffering.

### The Effects of 20-HETE on the Phosphorylation of ERK1/2 and JNK, and on the Serine Phosphorylation of IRS-1

First of all, human umbilical vein endothelial cells (HUVECs) were cultured for 18 hours in serum-deprived medium and incubated for various concentrations of 20-HETE (1 nm, 2 nm, 5 nm, 10 nm). Human umbilical vein endothelial cells (HUVECs) were cultured for 18 hours in serum-deprived medium and incubated for various times in the presence or absence of 5 nmol/L 20-HETE. The MEK1 inhibitor PD98059 (50 nmol/L) or JNK inhibitor SP600125 (30 nm) was added to the cells 30 minutes before the addition of 20-HETE. Equal amounts of cell lysates were incubated with p-Ser^312^-IRS-1, p-Ser^616^-IRS-1, phospho-ERK1/2, or phospho-JNK antibodies. To normalize the blots for protein levels, immunoblotting was performed with anti-phosphospecific antibodies; the blots were then stripped and reprobed with anti-IRS-1, anti-ERK1/2, or anti-JNK antibody.

### The Effects of 20-HETE on the Tyrosine Phosphorylation of IRS-1, the Association of IRS-1 with the p85 Subunit of PI3-Kinase, and Akt and eNOS Phosphorylation

Human umbilical vein endothelial cells (HUVECs) were cultured for 18 hours in serum-deprived medium containing 10 mmol/L glucose and incubated for 30 minutes in the presence or absence of 5 nmol/L 20-HETE; the cells were then stimulated with 100 nmol/L insulin. PD98059 (50 nmol/L) was added to the cells 30 minutes before the addition of 20-HETE. Equal amounts of cell lysates were separately incubated with anti-Tyrosine-IRS-1, anti-PI3K(P85 subunit), p-Ser^473^-AKT, and p-Ser^1177^-eNOS. To normalize the blots for protein levels, immunoblotting was performed with anti-phosphospecific antibodies; the blots were then stripped and reprobed with IRS-1, AKT, eNOS, and β-actin antibodies.

### NOS activity and cGMP production

NOS activity was determined in whole-cell lysates of HUVECs using a NOS detection system (Sigma, Saint Louis, Mo, USA) according to the manufacturer's instructions; this system measures the ability of NOS to convert L-[^14^C] arginine to L-[^14^C] citrulline. The data were normalized based on the amount of protein used and the reaction time. The amount of cyclic GMP production in the medium by the HUVECs was assessed by a cGMP assay kit (Cayman, Ann Arbor, MI) according to the manufacturer's instructions.

### In vivo insulin stimulation

Based on results from a previous study and preliminary studies [19], the mice were injected with 20-HETE (10 mg/kg body weight) via the tail vein 1 h before insulin injection. In experiments using MEK inhibitor PD98059, the substance was injected i.p. at 10 mg/kg of body weight 2 h before 20-HETE injection. The animals were then stimulated with insulin (i.p., 0.75 mU/g of body weight) and euthanized with sodium pentobarbital (40 mg/kg of body weight). Aortas were immediately collected and snap-frozen in liquid nitrogen. Insulin signaling studies were then performed on aortic lysates as described above.

### Assessment of Insulin-induced Vasorelaxation

The insulin-induced aortic rings relaxation experiments were performed according to a previously described method [20], [21]. Briefly, insulin (10^−8^–10^−5^ M) was added in a cumulative manner. Arteries were incubated in the presence and absence of *N* ^G^-nitro-L-arginine (L-NNA; 10^−4^ M), 20-HETE (1 µM) and PD98059 (10 µM). For insulin curves, relaxation at each concentration was measured and expressed as percent maximum relaxation, where 100% is equivalent to loss of all tension developed in response to phenylephrine.

### Immunoblotting

The protein contents of extracts were determined using the Bradford method. Extracts were resolved using SDS-PAGE and transferred to polyvinylidene difluoride (PVDF) filter membranes. Proteins were detected by immunoblotting and visualized using enhanced chemiluminescence. Procedures were performed as described previously [22].

### Statistical analysis

The data are presented as the means ± SEM. Comparisons among groups were performed using one-way ANOVA, followed by a Scheffe F-test. *P*<0.05 was accepted as statistically significant.

## Results

### 20-HETE-induced Phosphorylation of ERK1/2 and Site-Specific Serine Phosphorylation of IRS-1

**Figure 1:**
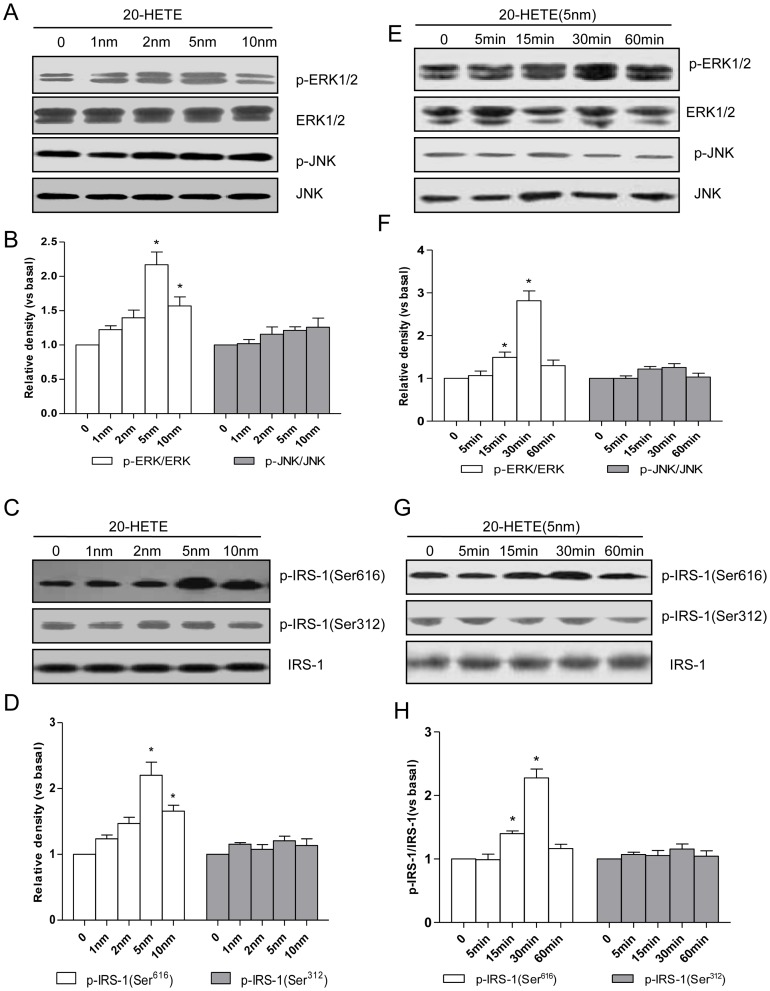
Concentration and time course of the effects of 20-HETE on JNK phosphorylation, ERK1/2 phosphorylation, and IRS-1 Serine phosphorylation in HUVECs. (A) A representative immunoblot of ERK1/2 phosphorylation and JNK phosphorylation induced by different concentration of 20-HETE; (B) Statistical analysis of the phosphorylation of ERK1/2 and JNK in Figure 1A; (C) A representative immunoblot of the Ser^616^ and Ser^312^ phosphorylation of IRS-1 induced by different concentration of 20-HETE; (D) Statistical analysis of the phosphorylation of IRS-1 at Ser^616^ and Ser^312^ in Figure 1C; (E) A representative immunoblot of ERK1/2 phosphorylation and JNK phosphorylation induced by 20-HETE at different time; (F) Statistical analysis of the phosphorylation of ERK1/2 and JNK in Figure 1E; (G) A representative immunoblot of the Ser^616^ and Ser^312^ phosphorylation of IRS-1 induced by 20-HETE at different time; (H) Statistical analysis of the phosphorylation of IRS-1 at Ser^616^ and Ser^312^ in Figure 1G;Each bar represents the mean ± SD of three independent experiments; *: *P*<0.05. measurements in 20-HETE-treated cells versus measurements in non-20-HETE-treated cells.

**Figure 2:**
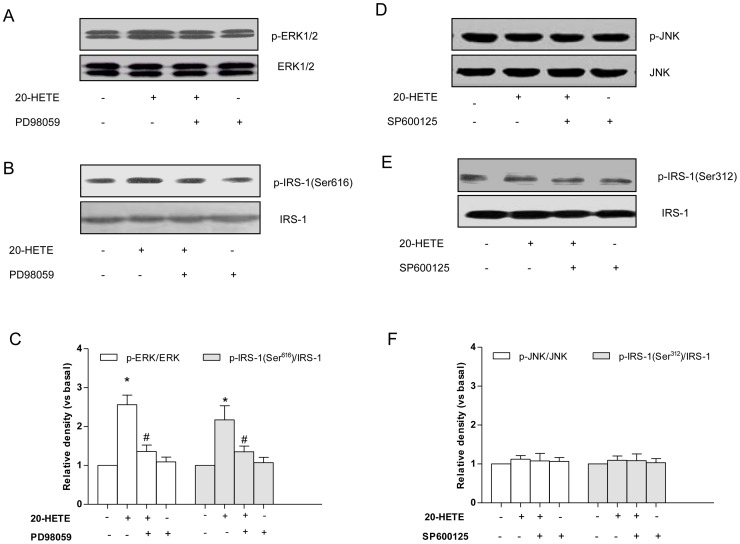
The effects of the ERK1/2 inhibitor on 20-HETE-stimulated IRS-1 Serine phosphorylation and ERK1/2 phosphorylation in HUVECs. PD98059 (50 nmol) or SP600125 (20 nm) was added to the cells 30 min before the addition of 20-HETE. (A) A representative immunoblot of ERK1/2 phosphorylation; (B) A representative immunoblot of the Ser^616^ phosphorylation of IRS-1; (C) Statistical analysis of the phosphorylation of ERK1/2 and the phosphorylation of IRS-1 at Ser^616^; (D) A representative immunoblot of JNK phosphorylation; (E) A representative immunoblot of the Ser^312^ phosphorylation of IRS-1; (F) Statistical analysis of the phosphorylation of JNK and the phosphorylation of IRS-1 at Ser^312^. Each bar represents the mean ± SD of three independent experiments; *: *P*<0.05. measurements in 20-HETE-treated cells versus measurements in non-20-HETE-treated cells. #: *P*<0.05. measurements in PD98059-treated cells versus measurements in 20-HETE-treated cells.

Increased serine phosphorylation of IRS-1 has been shown to inhibit the ability of IRS-1 to be tyrosine phosphorylated by the insulin receptor and to bind and activate PI3-kinase. Specifically, it has been shown that this function is performed by ERK1/2 at Ser^616^ of IRS-1. As shown in the present study, 20-HETE treatment caused a dose-dependent increase in the phosphorylation of ERK1/2 but not JNK in HUVECs (Fig. 1A and B), with the maximal effects occurring at 5 nm. These stimulatory effects of 20-HETE were accompanied by time-dependent increases in IRS-1 phosphorylation at Ser^616^ but not at Ser^312^ (Fig. 1C and D). Additionally, 20-HETE treatment also induced a time-dependent increase in the phosphorylation of ERK1/2 but not JNK in HUVECs (Fig. 1E and F), with the maximal effects occurring after 30 min of incubation. These stimulatory effects of 20-HETE were accompanied by time-dependent increases in IRS-1 phosphorylation at Ser^616^ but not at Ser^312^ (Fig. 1G and H). The ERK1/2 inhibitor PD98059 or JNK inhibitor SP600125 (30 nm) was added to the cells 30 minutes before 20-HETE addition. The results showed that PD98059 blocked 20-HETE-induced phosphorylation of ERK1/2 (Fig. 2A and C; *P*<0.05). Consequently, the 20-HETE-induced phosphorylation of IRS-1 at Ser^616^ was also reversed by treatment with PD98059 (Fig. 2B and C; *P*<0.05). However, 20-HETE or SP600125 does not affect the phosphorylation of JNK and IRS-1 phosphorylation at Ser^312^ (Fig. 2D, E and F).

### 20-HETE impaired Insulin-Stimulated Tyrosine Phosphorylation of IRS-1 and PI3K/Akt activation

**Figure 3:**
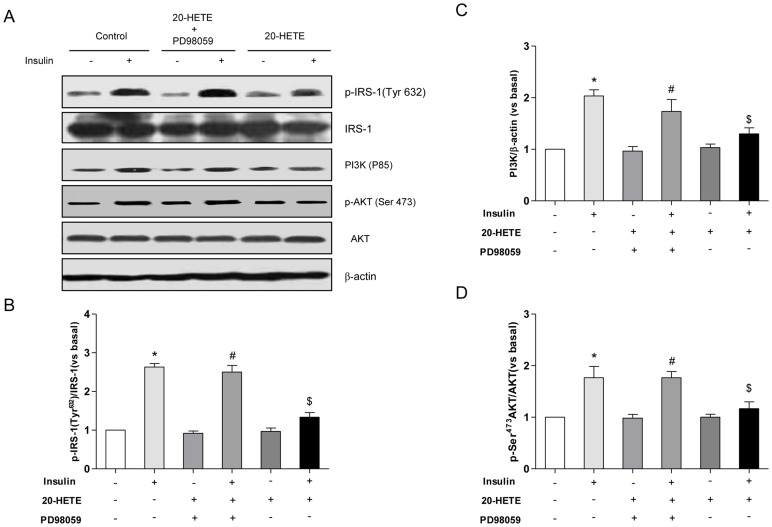
The effects of 20-HETE on the insulin-stimulated tyrosine phosphorylation of IRS-1 and on PI3K/Akt activation. HUVECs were treated with 20-HETE in the presence or absence of PD98059, and 100 nmol insulin was then added for 10 min. (A) A representative immunoblot of the Tyr^632^ phosphorylation of IRS-1; PI3K and the Ser^473^ phosphorylation of Akt (B) Statistical analysis of the tyrosine phosphorylation of IRS-1; (C) Statistical analysis of P13K(p85) expression; (D) Statistical analysis of the Ser^473^ phosphorylation of Akt. Each bar represents the mean ± SD of three independent experiments; *: *P*<0.05, versus control; #: *P*<0.05, versus 20-HETE + PD98059; $: *P*<0.05, versus insulin.

Because the serine phosphorylation of IRS-1 converts IRS-1 into an inhibitor of the intrinsic IR tyrosine kinase, we tested the possibility that the increased Ser616 phosphorylation of IRS-1 induced by 20-HETE would be associated with impaired insulin-stimulated tyrosine phosphorylation of IRS-1. As shown in Figure 3A, exposure of HUVECs to 20-HETE resulted in an approximately 30% inhibition of insulin-stimulated Tyr632 phosphorylation of IRS-1. The inhibitory effect of 20-HETE was reversed by treatment with PD98059. Because the association of PI3K/Akt with tyrosine-phosphorylated IRS-1 is essential for downstream insulin signaling, the effect of 20-HETE on IRS-1/p85/Akt docking was examined by immunoblotting with anti-p85 and anti-Ser473 Akt antibodies. As shown in Figure 3C and D, respectively, insulin increased the binding of IRS-1 to the p85 subunit 1.8-fold and increased Ser473 Akt phosphorylation 1.6-fold. Treatment with 20-HETE decreased the insulin-stimulated binding of IRS-1 to the p85 subunit by 30% and decreased Ser473 Akt activation by 40%. These inhibitory effects of 20-HETE were reversed by PD98059. Besides that, both ERK1/2 and JNK were phosphorylated by insulin treatment, which were accompanied by IRS-1 phosphorylation at Ser616 and Ser 312(Figure S1 and S2). However, 20-HETE treatment did not reinforce these stimulatory effects of insulin (Figure S1 and S2).

### 20-HETE Impaired the Insulin-stimulated Activation of eNOS and eNOS Activity

**Figure 4:**
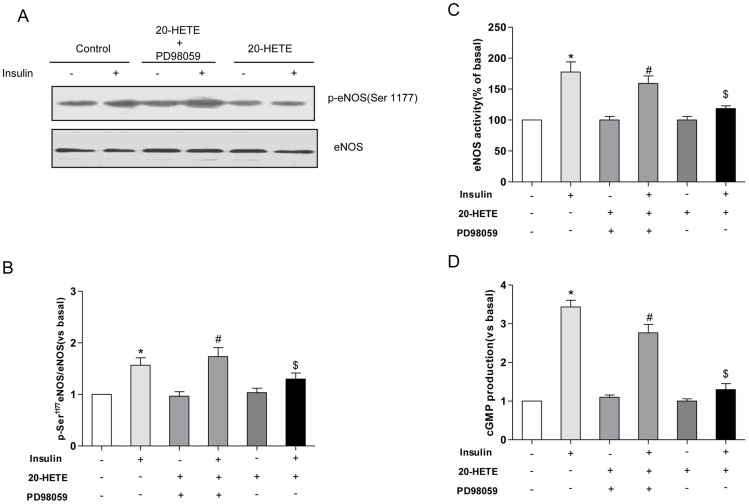
The effects of 20-HETE on the insulin-stimulated activation of eNOS and eNOS activity. HUVECs were treated as indicated in Figure 3. (A) A representative immunoblot of the phosphorylation of eNOS at Ser^1177^; (B) Statistical analysis of the phosphorylation of eNOS at Ser^1177^; (C) NOS activity was determined in cell lysates of HUVECs using a NOS detection system according to the manufacturer's instructions; (D) The release of NO into the medium by HUVECs was assessed by measuring cGMP level. Each bar represents the mean ± SD of three independent experiments; *: *P*<0.05, versus control; #: *P*<0.05, versus 20-HETE + PD98059; $: *P*<0.05, versus insulin.

Insulin increased the phosphorylation of eNOS on Ser^1177^ 1.6-fold (Figure 4A and B). Treatment with 20-HETE decreased insulin-stimulated Ser^1177^ eNOS phosphorylation by 30% (based on the levels in 20-HETE-treated, insulin-stimulated cells versus the levels in non-20-HETE-treated, insulin-stimulated cells; *P*<0.05). Treatment of HUVECs with PD98059 reversed the inhibitory effect of 20-HETE (Figure 4A and B, *P*<0.05). We next determined whether 20-HETE would affect the eNOS activation and cGMP production induced by insulin. Insulin stimulated eNOS activity 1.7-fold whereas 20-HETE treatment decreased insulin-stimulated eNOS activity by 30% (Figure 4C; *P*<0.05). Treatment of HUVECs with PD98059 partially reversed the inhibitory effects of 20-HETE on insulin-stimulated eNOS activity (Figure 4C; *P*<0.05). NO production was reflected by measuring cGMP levels, insulin stimulated cGMP production 3.5-fold, whereas 20-HETE treatment decreased of insulin-stimulated cGMP production by 75% (Figure 4D; *P*<0.05). Treatment of HUVECs with PD98059 partially reversed the inhibitory effects of 20-HETE on insulin-stimulated cGMP production (Figure 4D).

### 20-HETE Induced Serine Phosphorylation of IRS-1 in C57BL/6J Mouse Aortas

**Figure 5:**
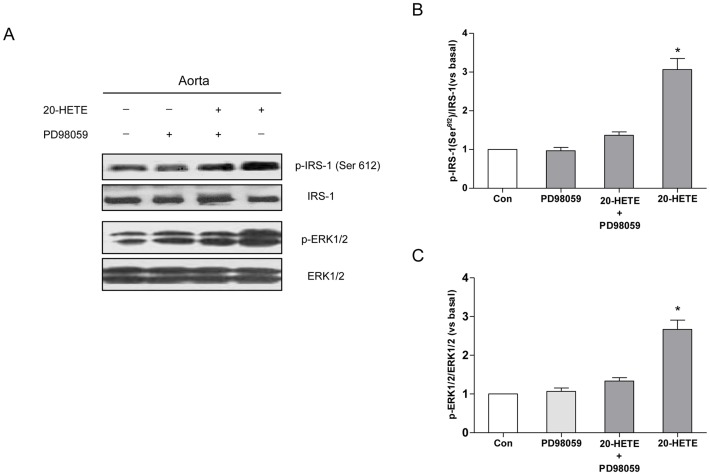
The effects of 20-HETE on the serine phosphorylation of IRS-1 in C57BL/6J mouse aortas. (A) A representative immunoblot of the Ser^616^ phosphorylation of IRS-1 and of ERK1/2 phosphorylation; (B) Statistical analysis of the phosphorylation of IRS-1 at Ser^616^; (C) Statistical analysis of ERK1/2 phosphorylation. Each bar represents the mean ± SD of three independent experiments; *: *P*<0.05, measurements in 20-HETE-treated aorta versus measurements in non-20-HETE-treated aorta.

In mice treated for 1 h with 20-HETE, the phosphorylation of IRS-1 at Ser^612^ (orthologous to Ser^616^ in human IRS-1) in the aortas was increased 3.2-fold (Figure 5A and B; *P*<0.05). These stimulatory effects of 20-HETE were accompanied by increased phosphorylation of ERK1/2 (Figure 5A and C; *P*<0.05). These effects of 20-HETE were reversed by treatment with PD98059 (Figure 5A, B, and C; *P*<0.05).

### 20-HETE Impaired Insulin-stimulated Activation of the Tyrosine Phosphorylation of IRS-1, PI3-kinase, Akt, and eNOS C57BL/6J Mouse Aortas

**Figure 6:**
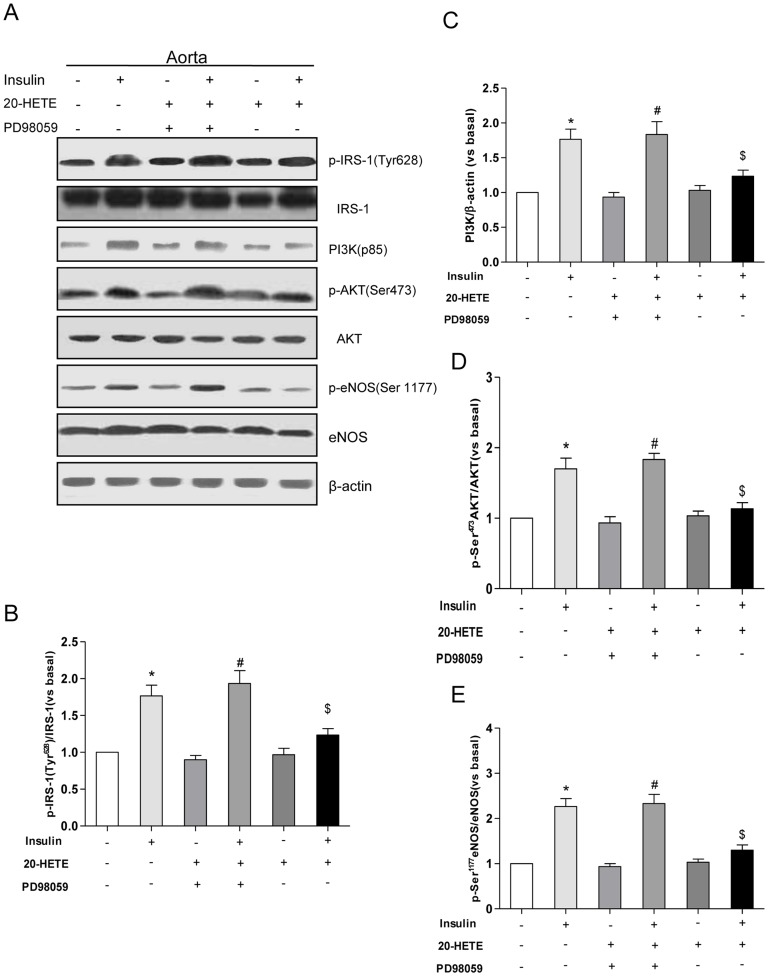
The effects of 20-HETE on the insulin-stimulated activation of the tyrosine phosphorylation of IRS-1, PI3K, Akt, and eNOS in C57BL/6J mouse aortas. (A) A representative immunoblot of the Tyr^628^ phosphorylation of IRS-1, p85, Ser^473^-AKT, and Ser^1177^-eNOS; (B) Statistical analysis of the tyrosine phosphorylation of IRS-1; (C) Statistical analysis of P13K(p85) expression; (D) Statistical analysis of the Ser^473^ phosphorylation of Akt; (E) Statistical analysis of the Ser^1177^ phosphorylation of eNOS. Each bar represents the mean ± SD of three independent experiments; *: *P*<0.05, versus control; #: *P*<0.05, versus 20-HETE + PD98059; $: *P*<0.05, versus insulin.

We tested whether 20-HETE would also affect the activation of the insulin-stimulated IRS-1/PI3KAkt/eNOS pathway in vivo in mice treated for 1 h with 20-HETE. Insulin increased the Tyr^628^ phosphorylation of IRS-1 (corresponding to Tyr^632^ in human IRS-1) and PI3K activation (Figure 6A, B and C; *P*<0.05). Insulin stimulated Akt phosphorylation on Ser^473^ 1.6-fold and the phosphorylation of eNOS on Ser^1177^ 2-fold in the mouse aortas (comparing the levels in the aortas of insulin-stimulated mice versus basal levels; Figure 6A, D, and E; *P*<0.05). Treatment with 20-HETE resulted in 40 and 50% decreases of insulin-stimulated Akt and eNOS phosphorylation, respectively (Figure 6A, D, and E; *P*<0.05). Treatment of mice with PD98059 reversed the inhibitory effect of 20-HETE (Figure 6A, D, and E; *P*<0.05).

### 20-HETE Inhibited Insulin-induced Vasodilation

To evaluate endothelial function, the vasorelaxation responses to insulin were examined in mouse aortas. Insulin-induced concentration-dependent relaxations of aortic rings were obtained from the C57BL/6 mice, and the relaxations were significantly inhibited in the presence of either L-NNA (inhibitor of NOS) or 20-HETE, but PD98059 (inhibitor of ERK1/2) blocked the inhibitory effect of 20-HETE significantly (Figure S3).

## Discussion

In this study, we inquired whether 20-HETE-induced alterations in insulin signaling contribute to an impairment of endothelial insulin action. The current study is the first to provide strong direct evidence that exposure of HUVECs to 20-HETE inhibited insulin-stimulated NOS activity and NO production. This event was associated with an increased ERK1/2 activity that was paralleled by increased IRS-1 phosphorylation at Ser^616^ and decreased insulin-stimulated IRS-1 tyrosine phosphorylation. This study is potentially of great significance, because it brings into being a new face to a prominent eicosanoid and suggests a feed-forward amplification of endothelial dysfunction and insulin resistance induced by 20-HETE.

It is well accepted that the serine phosphorylation of IRS-1 is a negative regulator of insulin signaling [23]. ERK1/2 regulates stress responses, inflammation, and apoptosis, and has been implicated in the pathogenesis of endothelial dysfunction and insulin resistance [24]. ERK1/2 activation has been shown to result in an increased phosphorylation at Ser^612^ of IRS-1 (orthologous to Ser^616^ in human IRS-1), thereby inhibiting the insulin-stimulated tyrosine phosphorylation of IRS-1 and subsequent activation of PI3K [25]. Because 20-HETE activates ERK1/2 in cultured vascular endothelial cells [17], [26], we examined the possibility that the 20-HETE-induced phosphorylation at Ser^616^ of IRS-1, which is mediated by ERK1/2, may account for the inhibitory effects of 20-HETE on the insulin signaling pathway that is involved in NO production. Similar to previous researchers [17], we found that HUVECs exhibited increased ERK1/2 activity in a time-dependent manner when exposed to 20-HETE. Interestingly, IRS-1 phosphorylation at Ser^616^ was increased with a concomitant increase in ERK1/2 activity. The specific inhibitor of ERK1/2, PD98059, completely abolished 20-HETE-induced IRS-1 serine phosphorylation and ERK1/2 phosphorylation. These data suggest that 20-HETE may induce ERK1/2-dependent endothelial insulin resistance and dysfunction. The vasodilatory effects of insulin are mediated by the IRS-1/PI-3 kinase/Akt/eNOS signaling pathway that leads to increased endothelial NO production. A growing body of evidence indicates that the serine phosphorylation of IRS-1, which is induced by a variety of factors, interferes with the ability of IRS-1 to be tyrosine-phosphorylated upon insulin stimulation and reduces its ability to engage the p85 subunit of PI 3-kinase [27], [28]. Our findings demonstrate that the exposure of HUVECs to 20-HETE results in decreased NO production upon insulin stimulation and is associated with impairment of tyrosine phosphorylation of IRS-1 and its corresponding association with the p85 subunit of PI 3-kinase, leading to the defective activation of Akt and eNOS.

The phosphorylation of eNOS (at Ser1177 in humans) and its association with 90-kDa heat shock protein (HSP90) are both required for eNOS activation [29]. In primary human endothelial cells, previous studies show that impairment of the insulin signaling involving IRS-1/PI3-kinase/Akt induced by either Ang II or IL-6 in IRS-1 resulted in a decrease in the ability of insulin to phosphorylate eNOS at the positive regulatory site Ser1177 and to dephosphorylate the inhibitory site Thr495 [18], [30]. Accordingly, the present study demonstrated that the impaired activation of the insulin-dependent IRS-1/PI3-kinase/Akt pathway in HUVECs exposed to 20-HETE was associated with a decrease in Ser1177phosphorylation, resulting in a reduction in NOS activity. These findings are in contrast to those reported in bovine aortic endothelial cells (BAECs), in which the addition of 5 nM 20-HETE (the same concentration that used in the current study) had no effect on the basal and ionophore-stimulated levels of eNOS phosphorylation at either Ser1179 or Thr497 but inhibited the association of eNOS with HSP90 [10]. This disparity might have arisen because the cells are different in that they originate from different vascular beds that respond to 20-HETE with opposing outcomes.

Additionally, as demonstrated in this study, the inhibition of ERK1/2 activity with its specific inhibitor PD98059 completely abolished 20-HETE-induced IRS-1 serine phosphorylation and restored the insulin sensitivity of IRS-1 tyrosine phosphorylation, Akt phosphorylation, and eNOS activity in the presence of 20-HETE. This strongly suggests that 20-HETE induces endothelial cell insulin resistance via an ERK1/2-dependent pathway.

The results obtained in aortas isolated from C57BL/6J mice support the likelihood that our findings in cultured endothelial cells may have physiological relevance. These in vivo findings demonstrate that 20-HETE induces phosphorylation at Ser^612^ of IRS-1 mediated by ERK1/2 and impairs the ability of insulin to stimulate eNOS Ser^1177^ phosphorylation via blunting the IRS-1/PI3-kinase/Akt pathway. Insulin-stimulated vasodilation were also attenuated by 20-HETE significantly, suggesting that 20-HETE impaired the endothelial function by terminating insulin signaling.

A close relationship between chronic inflammation and insulin resistance has been well established, and the IKKβ pathway is a target for insulin resistance [31]; in addition, IκB was activated by 20-HETE (data not shown), which is consistent with pervious observations that 20-HETE stimulates NFκB activation and promotes proinflammatory cytokine production via an ERK1/2-dependent pathway [17]. Additionally, 20-HETE increases superoxide production, activates NAPDH oxidase in endothelial cells and promotes the development of vascular dysfunction [32], [33]. Oxidative stress plays a pivotal role in the development of diabetes complications, both microvascular and cardiovascular in origin. The metabolic abnormalities of diabetes cause mitochondrial superoxide overproduction in the endothelial cells of both large and small vessels, as well as in the myocardium [34]. Therefore, the proinflammatory and pro-oxide stress effects of 20-HETE on endothelial cells may exacerbate endothelial insulin resistance and dysfunction.

20-HETE is believed to be a prohypertensive eicosanoid, and inhibition of 20-HETE in experimental models of hypertension reduces blood pressure [35]. Clinic evidence has indicated a significant positive association between 20-HETE excretion and BMI [36], and obesity has been linked with insulin resistance [37], which may be a rational explanation for the inhibitory effect of 20-HETE on insulin vascular action. Based on the results, 20-HETE production and/or action might be a therapeutic target to treat hypertension associated with insulin resistance states. Taken together, these data support the concept that 20-HETE impairs the ability of insulin to stimulate NOS activity by regulating the reciprocal phosphorylation of eNOS at Ser^1177^ via mechanisms involving the activation of PI3-kinase/Akt. The inhibitory effects of 20-HETE are likely to be mediated by the activation of ERK1/2, which induces the phosphorylation of IRS-1 at Ser^616^, leading to the dysfunction of IRS-1 as a docking protein. This provides support for the hypothesis that 20-HETE may play an important role in the pathophysiology of cardiovascular disease associated with hypertension and insulin resistance.

## Supporting Information

Figure S1 **The effects of 20-HETE on the insulin-stimulated JNK phosphorylation, ERK1/2 phosphorylation, and IRS-1 Serine phosphorylation in HUVECs.** HUVECs were treated with 20-HETE in the presence or absence of PD98059, and 100 nmol insulin was then added for 10 min. Insulin stimulates phosphorylation of ERK1/2 and JNK. (A) A representative immunoblot of ERK1/2 phosphorylation and JNK phosphorylation induced by different concentration of 20-HETE; (B) Statistical analysis of the phosphorylation of ERK1/2 in Figure S1-A; (C) Statistical analysis of the phosphorylation of JNK in Figure S1-A. Each bar represents the mean ± SD of three independent experiments; *: *P*<0.05, versus control.
(TIF)

Figure S2 **The effects of 20-HETE on the insulin-stimulated IRS-1 Serine phosphorylation in HUVECs.** HUVECs were treated with 20-HETE in the presence or absence of PD98059, and 100 nmol insulin was then added for 10 min. Insulin induced IRS-1 phosphorylation at Ser^616^ and at Ser^312^. (A) A representative immunoblot of the phosphorylation of IRS-1 at Ser^312^ and Ser^616^; (B) Statistical analysis of the phosphorylation of JNK and the phosphorylation of IRS-1 at Ser^616^ in Figure S2-A; (C) Statistical analysis of the phosphorylation of IRS-1 at Ser^312^ in Figure S2-A. Each bar represents the mean ± SD of three independent experiments; *: *P*<0.05, versus control.
(TIF)

Figure S3 **The effects of 20-HETE on the insulin-induced vasorelaxation.** Insulin-induced concentration-dependent relaxations of aortic rings, but the relaxations were significantly inhibited in the presence of either L-NNA (inhibitor of NOS) or 20-HETE, but PD98059 (inhibitor of ERK1/2) reversed the inhibitory effect of 20-HETE. *: *P*<0.05, measurements in insulin + L-NNA versus control (insulin); #: *P*<0.05, measurements in insulin +20-HETE versus control (insulin); &: *P*<0.05, measurements in insulin +20-HETE +PD98059 versus insulin +20-HETE.
(TIF)
