# Supplementary figures and images for: 20-Hydroxyeicosatetraenoic Acid Impairs Endothelial Insulin Signaling by Inducing Phosphorylation of the Insulin Receptor Substrate-1 at Ser616
Source: PLoS One. 2014 Apr 24;9(4):e95841. doi: 10.1371/journal.pone.0095841 (PMC3998975; doi:10.1371/journal.pone.0095841)

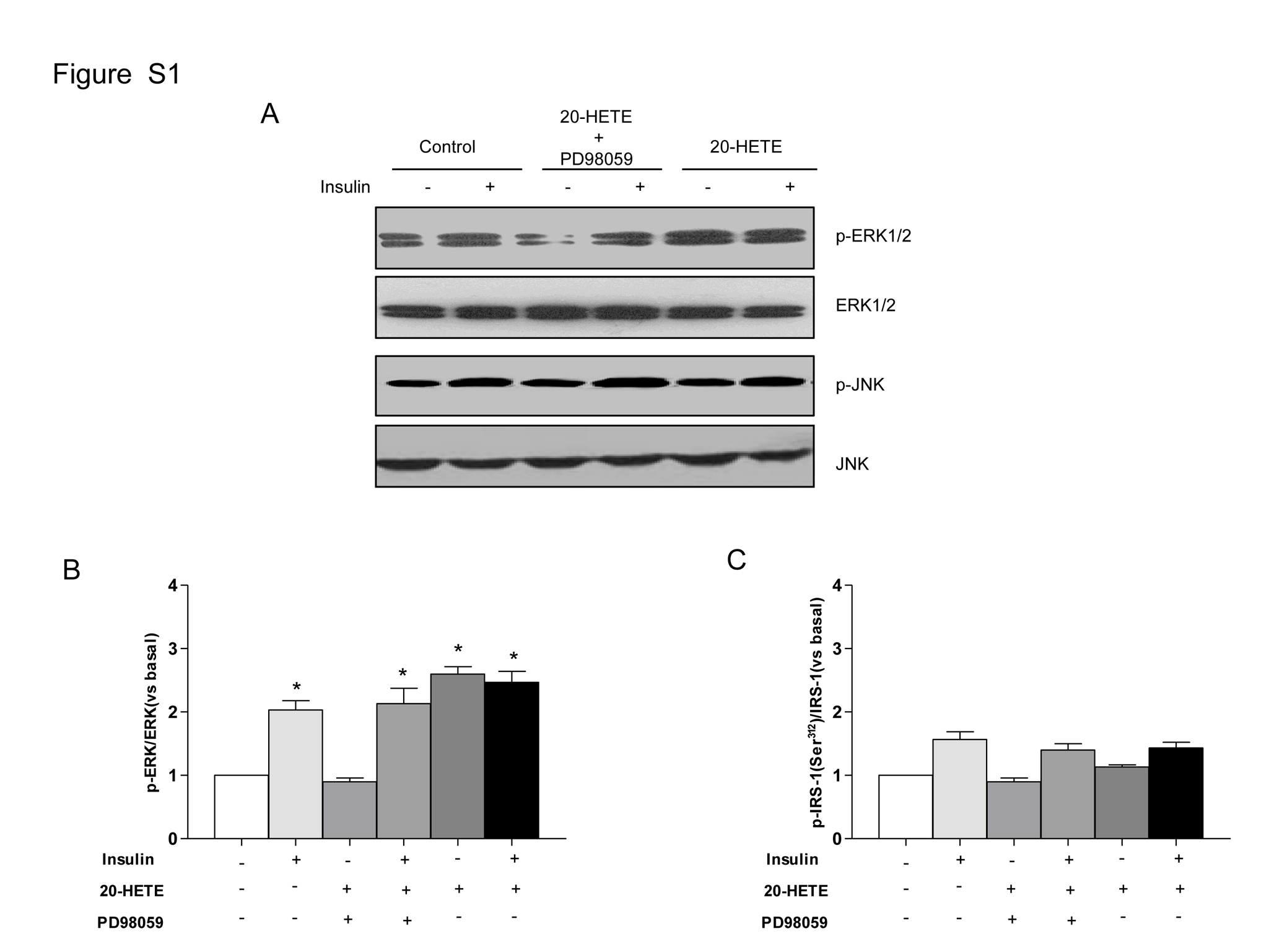

Supplement: Figure S1 — The effects of 20-HETE on the insulin-stimulated JNK phosphorylation, ERK1/2 phosphorylation, and IRS-1 Serine phosphorylation in HUVECs. HUVECs were treated with 20-HETE in the presence or absence of PD98059, and 100 nmol insulin was then added for 10 min. Insulin stimulates phosphorylation of ERK1/2 and JNK. (A) A representative immunoblot of ERK1/2 phosphorylation and JNK phosphorylation induced by different concentration of 20-HETE; (B) Statistical analysis of the phosphorylation of ERK1/2 in Figure S1-A; (C) Statistical analysis of the phosphorylation of JNK in Figure S1-A. Each bar represents the mean ± SD of three independent experiments; *: P<0.05, versus control. (TIF) [file pone.0095841.s001.tif]

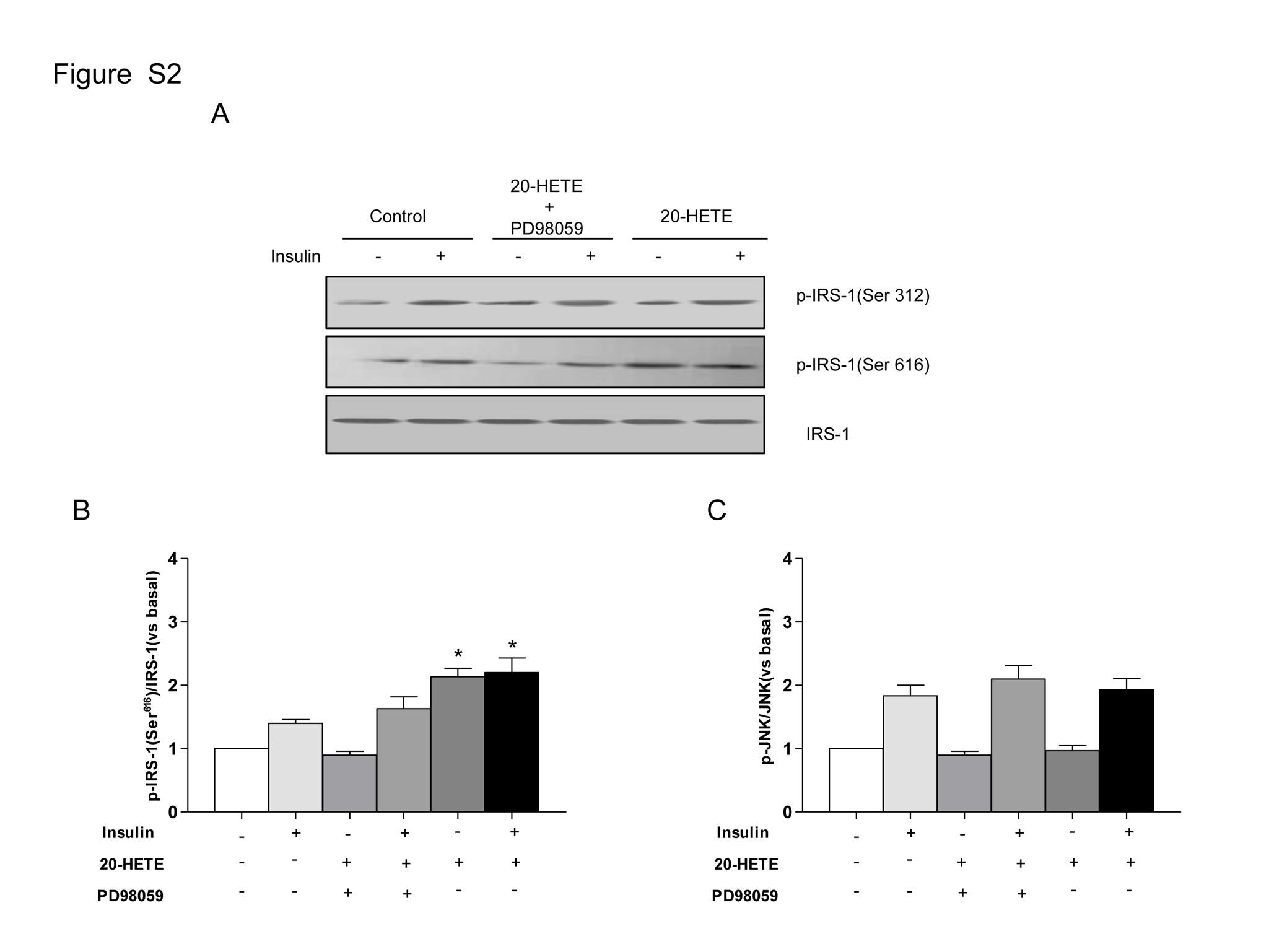

Supplement: Figure S2 — The effects of 20-HETE on the insulin-stimulated IRS-1 Serine phosphorylation in HUVECs. HUVECs were treated with 20-HETE in the presence or absence of PD98059, and 100 nmol insulin was then added for 10 min. Insulin induced IRS-1 phosphorylation at Ser616 and at Ser312. (A) A representative immunoblot of the phosphorylation of IRS-1 at Ser312 and Ser616; (B) Statistical analysis of the phosphorylation of JNK and the phosphorylation of IRS-1 at Ser616 in Figure S2-A; (C) Statistical analysis of the phosphorylation of IRS-1 at Ser312 in Figure S2-A. Each bar represents the mean ± SD of three independent experiments; *: P<0.05, versus control. (TIF) [file pone.0095841.s002.tif]

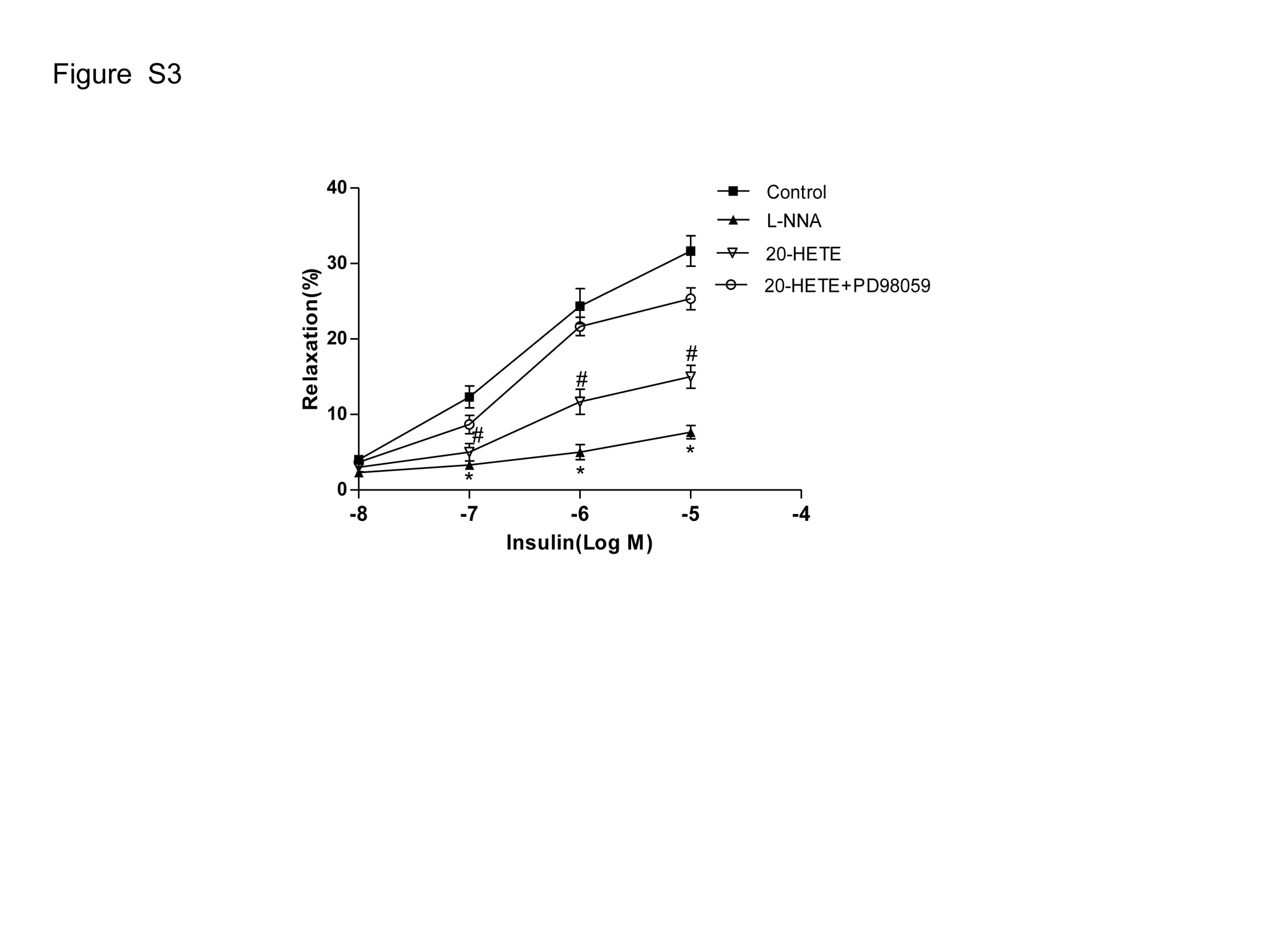

Supplement: Figure S3 — The effects of 20-HETE on the insulin-induced vasorelaxation. Insulin-induced concentration-dependent relaxations of aortic rings, but the relaxations were significantly inhibited in the presence of either L-NNA (inhibitor of NOS) or 20-HETE, but PD98059 (inhibitor of ERK1/2) reversed the inhibitory effect of 20-HETE. *: P<0.05, measurements in insulin + L-NNA versus control (insulin); #: P<0.05, measurements in insulin +20-HETE versus control (insulin); &: P<0.05, measurements in insulin +20-HETE +PD98059 versus insulin +20-HETE. (TIF) [file pone.0095841.s003.tif]
